# The effect of real-world and retinal motion on speed perception for motion in depth

**DOI:** 10.1371/journal.pone.0283018

**Published:** 2023-03-16

**Authors:** Yusei Yoshimura, Tomohiro Kizuka, Seiji Ono

**Affiliations:** 1 Graduate School of Comprehensive Human Sciences, University of Tsukuba, Ibaraki, Japan; 2 Faculty of Health and Sport Sciences, University of Tsukuba, Ibaraki, Japan; Justus Liebig Universitat Giessen, GERMANY

## Abstract

For motion in depth, even if the target moves at a constant speed in the real-world (physically), it would appear to be moving with acceleration on the retina. Therefore, the purpose of this study was to determine whether real-world and retinal motion affect speed perception in depth and to verify the influence of eye movements on both motion signals in judging speed in depth. We used a two-alternative forced-choice paradigm with two types of tasks. One stimulus moved at a constant speed in the real-world (world constant task) with three conditions: 80–60 cm (far), 60–40 cm (middle), and 40–20 cm (near) from the participant. The other stimulus moved at a constant speed on the retina (retinal constant task) with three conditions: 4–8 deg (far), 8–12 deg (middle), and 12–16 deg (near) as the vergence angle. The results showed that stimulus speed was perceived faster in the near condition than in the middle and far conditions for the world constant task, regardless of whether it was during fixation or convergence eye movements. In contrast, stimulus speed was perceived faster in the order of the far, middle, and near conditions for the retinal constant task. Our results indicate that speed perception of a visual target approaching the observer depends on real-world motion when the target position is relatively far from the observer. In contrast, retinal motion may influence speed perception when the target position is close to the observer. Our results also indicate that the effects of real-world and retinal motion on speed perception for motion in depth are similar with or without convergence eye movements. Therefore, it is suggested that when the visual target moves from far to near, the effects of real-world and retinal motion on speed perception are different depending on the initial target position.

## Introduction

It is crucial in our daily life to determine the speed of moving objects or people so that we do not collide when driving a car or walking on the street. It has been reported that speed perception involves signals from the retina, which are based on the vergence angle [1, 2]. When the target moves with constant speed in depth, the target moves with acceleration on the retina, unlike physical or two-dimensional (2D) motion. This is because the vergence angle increases exponentially as the target moves closer. Thus, when the target moves in three-dimension (3D), the target motion on the retina is not in accordance with the target motion in the real world. The question here is, which speed would we perceive, the motion on the retina or the motion in the real world when the target moves in 3D? A previous study has explored the difference in judgments of target speed discrimination between real-world (physical motion) and retinal motion in 3D motion. They have reported that there is no significant difference in speed change discrimination between the real world and retinal motion [3]. Another study has also reported that participants misperceive the acceleration of a visual target for motion in depth and overestimate the time to contact [4]. They suggest that participants do not use the acceleration information to estimate the time to contact. However, for motion in depth at different distances from the observer, it has not been fully clarified how real-world and retinal motion affects the speed perception of the target. Therefore, the first purpose of this study was to determine whether real-world and retinal motion affect speed perception for motion in depth.

It has also been reported that speed perception is determined based on information from extra-retinal signals in 2D motion [1, 5] or 3D motion [2, 6]. Thus, the effects of retinal and real-world motion on speed perception for motion in depth might be different between visual fixation, which is associated only with retinal signals, and eye movements, which produce retinal and extra-retinal signals. However, it is still uncertain whether convergence eye movements and fixation would result in retinal and real-world motion affecting speed perception differently, when the visual target is approaching the observer. Therefore, the second purpose of this study was to compare the influences of visual fixation and eye movements on speed perception in depth.

## Experiment 1: Comparison between the world and retinal constant tasks

### Methods

Nine participants (6 men and 3 women; 21.4 ± 1.8 years old) volunteered to take part in this experiment. They reported having normal or corrected to normal vision and no known neurological or oculomotor disorder. This study was conducted in accordance with the Declaration of Helsinki, and all protocols were approved by the Research Ethics Committee at the Faculty of Health and Sport Sciences, University of Tsukuba. Written informed consent was obtained from all subjects before their participation.

**Fig 1:**
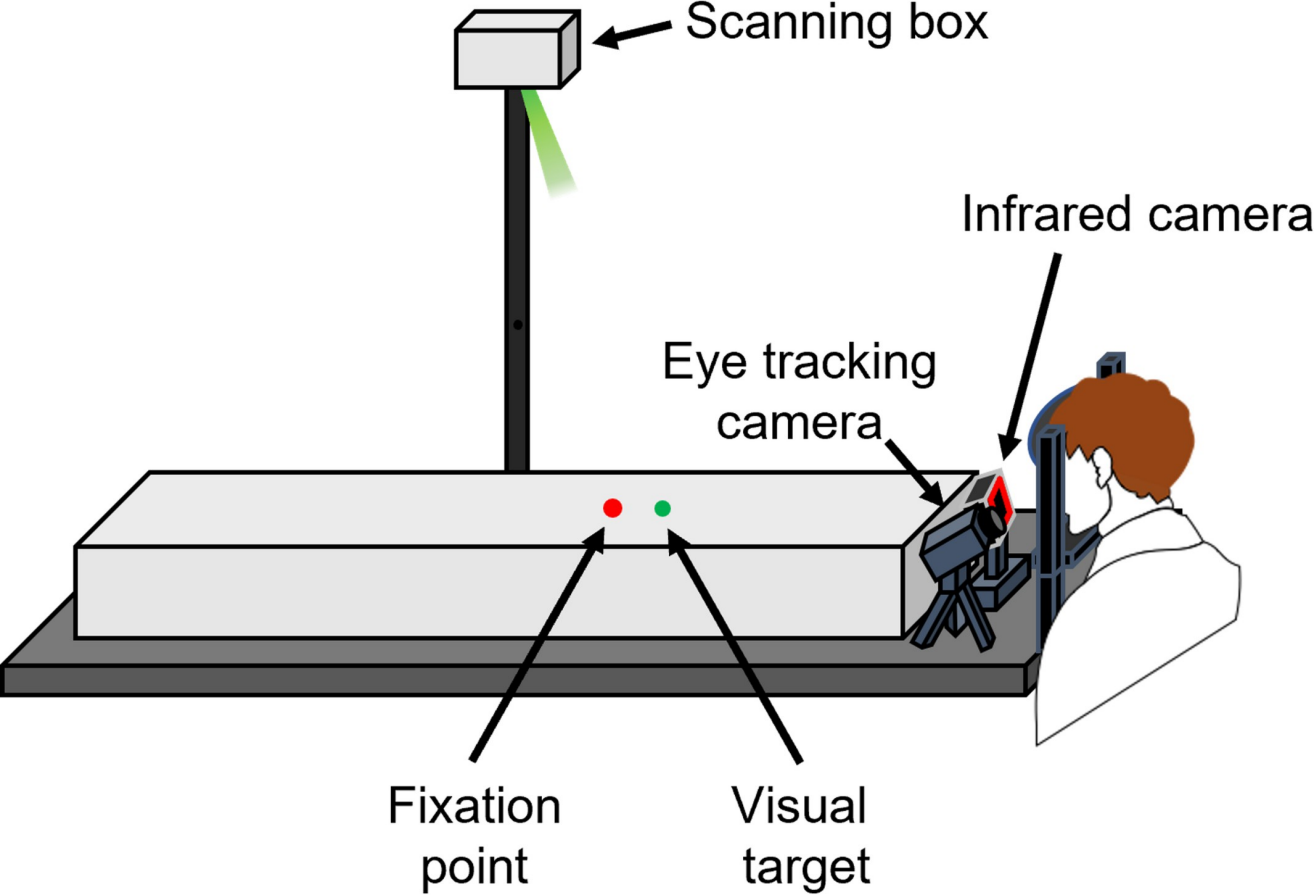
The experimental condition for speed perception tasks. Visual stimuli were moved from far to near and eye movements during the task were measured.

In this study, we measured speed perception using methods of psychophysics and eye movements. Fig 1 shows the experimental setup for speed perception tasks. The participants were seated with the head stabilized by a chin rest and a forehead restraint. The vertical distance from their eyes to the horizontal screen was 4 cm. The visual target was a green laser with a diameter of 2 mm, which was irradiated from the Scanning box (Novanta Japan Cor., TY) placed 200 cm above the horizontal screen. The target was controlled by Scan Master Designer and Scan Master Controller (Novanta Japan Cor., TY). The fixation point was a red laser with a diameter of 3 mm, which was irradiated from a self-built device, and was set at 4 cm behind the initial position of a visual target. Eye movements were measured by corneal reflex using an infrared camera to ensure whether participants were keeping the eye fixation or tracking the visual target with the eye, and eye position was detected by a video-based eye-tracking system [7–9]. The analog signals of eye and target positions were digitized at a sampling rate of 1000 Hz using an analog-to-digital converter system (Micro 1401–2, Cambridge Electronic Design Cor., Cambridge, UK).

**Fig 2:**
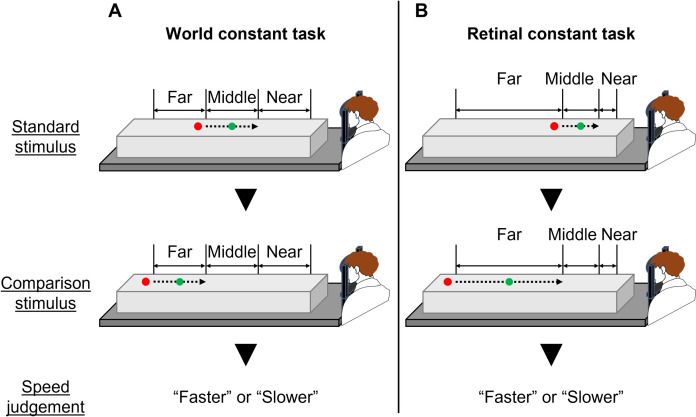
A two-alternative forced-choice paradigm. World constant task (A): the green circle (visual target) moved from far to near at constant velocity in the real world, while participants kept their eyes on the red circle (fixation point). Retinal constant task (B): visual target moved from far to near at constant velocity on the retina, while participants kept their eyes on the fixation point. In both speed perception tasks, participants compared the target speed between the standard and comparison stimuli.

To attain the first purpose, we conducted a world constant task and a retinal constant task using a two-alternative forced-choice (2AFC) paradigm to measure psychometric functions for speed perception. Fig 2 shows a schematic paradigm of 2AFC in each experimental task. Participants were presented with a sequence of stimuli and asked to respond by pressing a keyboard whether the speed of the latter stimulus (comparison stimulus) was perceived as faster or slower than the former stimulus (standard stimulus). Participants kept their gaze on a fixation point when presented with a sequence of stimuli for the world and retinal constant tasks. The trials, in which eye movements occurred during both tasks, were excluded. As visual stimuli, we used targets moving from far to near under three different conditions (far, middle and near conditions) based on different starting positions and five different target velocities. The standard stimulus was always moved in the middle condition and at the mean of five different target velocities. In contrast, the comparison stimulus was randomly moved under either far, middle or near conditions and at one of the five different target velocities. Each experimental task consisted of four blocks of 240 trials in total, with 16 trials at each comparison stimulus. Participants performed each task on two separate days considering the effects of their fatigue and burden.

**Fig 3:**
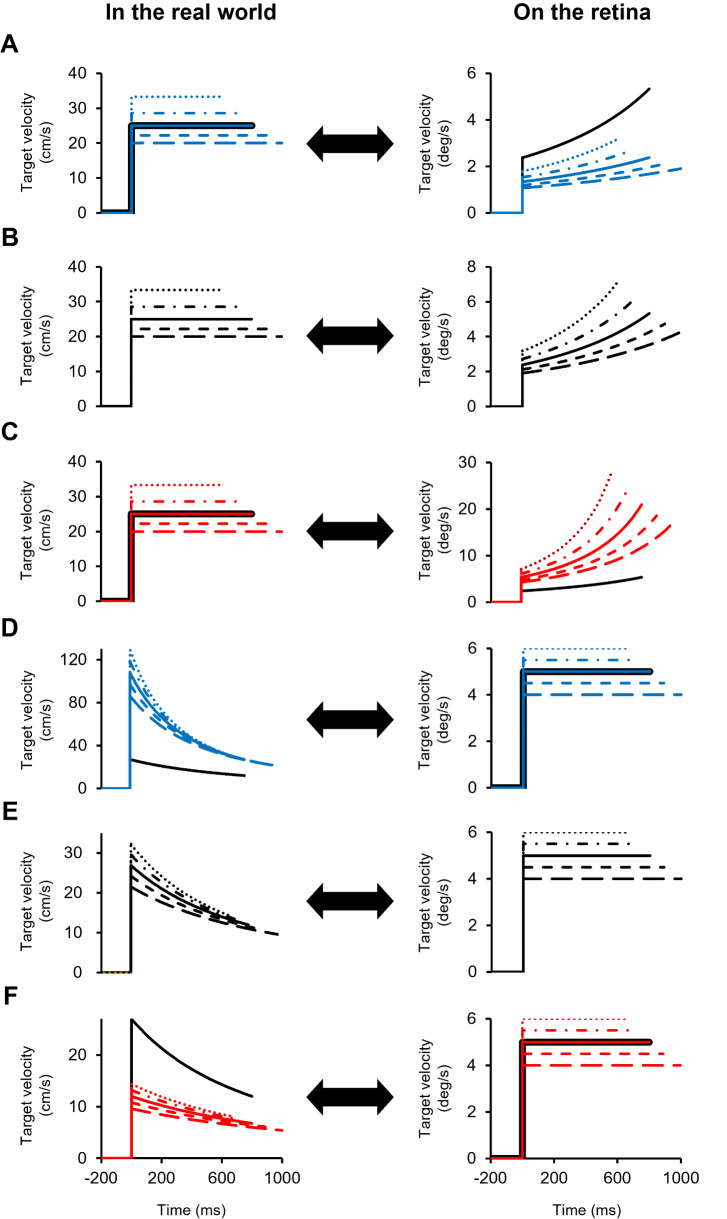
Target velocity of a speed perception task in the real world and on the retina. In the world constant task, the far (A), middle (B) and near (C) conditions are shown, and the target moves at a constant speed in the real world (physically), but it moves with acceleration on the retina. In the retinal constant task, the far (D), middle (E). and near (F) conditions are shown, and the target moves at a constant speed on the retina, but it moves with deceleration in the real world. The dotted, dashed-dotted, solid, short dashed and long dashed lines represent the order of speed. The blue, black and red lines indicate the far, middle and near conditions, respectively. The black solid lines indicate standard stimuli.

For the world constant task, the target moved 80 to 60 cm (far condition), 60 to 60 cm (middle condition) and 40 to 20 cm (near condition), which was equivalent to 4.3 to 5.7 deg (far condition), 5.7 to 8.6 deg (middle condition) and 8.6 to 17.1 deg (near condition) as the vergence angle. The target velocities were 20.0 cm/s (1000 ms), 22.2 cm/s (900 ms), 25.0 cm/s (800 ms), 28.6 cm/s (700 ms) and 33.3 cm/s (600 ms) (Fig 3A–3C).

For the retinal constant task, the target moved 4 to 8 deg (far condition), 8 to 12 deg (middle condition) and 12 to 16 deg (near condition) as the vergence angle, which was equivalent to 86 to 43 cm (far condition), 43 to 29 cm (middle condition) and 29 to 21 cm (near condition). The target velocities were 4.0 deg/s (1000 ms), 4.5 deg/s (889 ms), 5.0 deg/s (800 ms), 5.5 deg/s (727 ms) and 6.0 deg/s (667 ms) (Fig 3D–3F).

The probability that a participant indicated “Faster” for each velocity in the 2AFC paradigm was calculated on three different tasks. Psychometric functions using a binomial logistic regression analysis were also derived from the probability in three different conditions. If the statistical significance level of the model obtained from the analysis was *p* < 0.05 and the goodness of fit of the model was *p* ≥ 0.05, the point of subjective equality (PSE) was calculated based on the psychometric function. In this study, we defined PSE as the speed of the 50% point on the fitted psychometric function.

The position data of convergence eye movements, which are disconjugate eye movements evoked by a visual target moving from far to near, were derived by subtracting the right eye position from the left eye position. Convergence eye velocity was generated by digital differentiation of the position arrays using a central difference algorithm in MATLAB (Mathworks, MA). The onset of smooth convergence eye movements was taken as the time that the average eye velocity reached >3 times standard deviation (SD) above the pretrial values during fixation.

We conducted the paired t-test in order to compare the speed perception between three different conditions in each experimental task. The effect size was calculated as *r*. *r* was defined as small when *r* < 0.3, medium when 0.3 ≤ *r* < 0.5, and large when 0.5 ≤ *r*. The method of Holm was used to adjust the *p* values in multiple testing [10]. All statistical analyses were run by using SPSS ver. 28.0 (IBM Cor., NY), and the level of statistical significance was *p* < 0.05.

### Results

**Fig 4:**
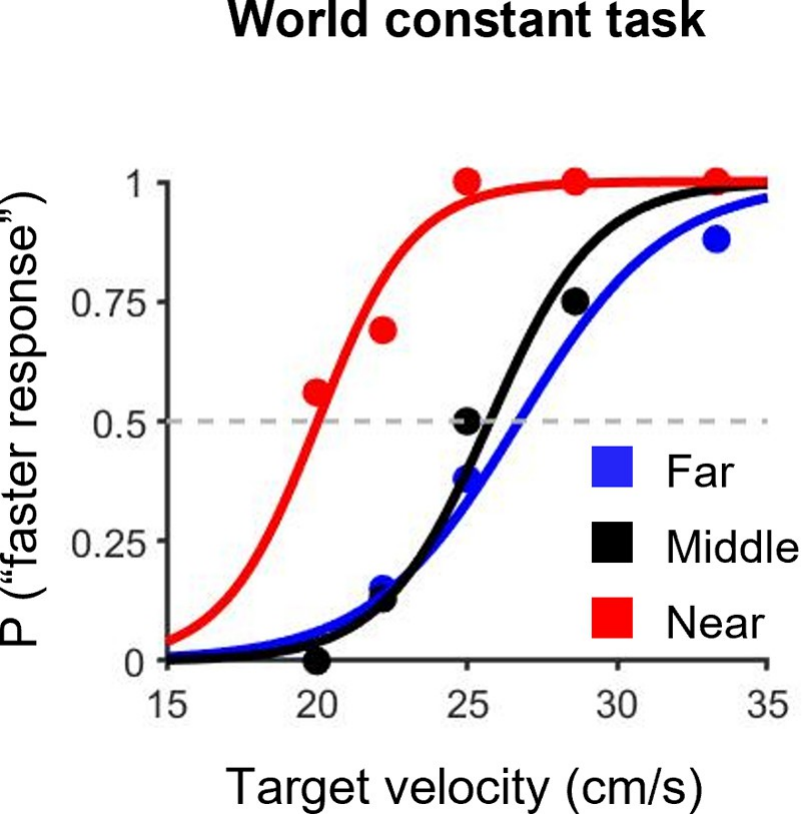
A typical example of psychometric functions for the world constant task. Colors indicate the three different initial positions of the target from the participants: blue, black and red lines are the far, middle and near conditions, respectively.

**Fig 5:**
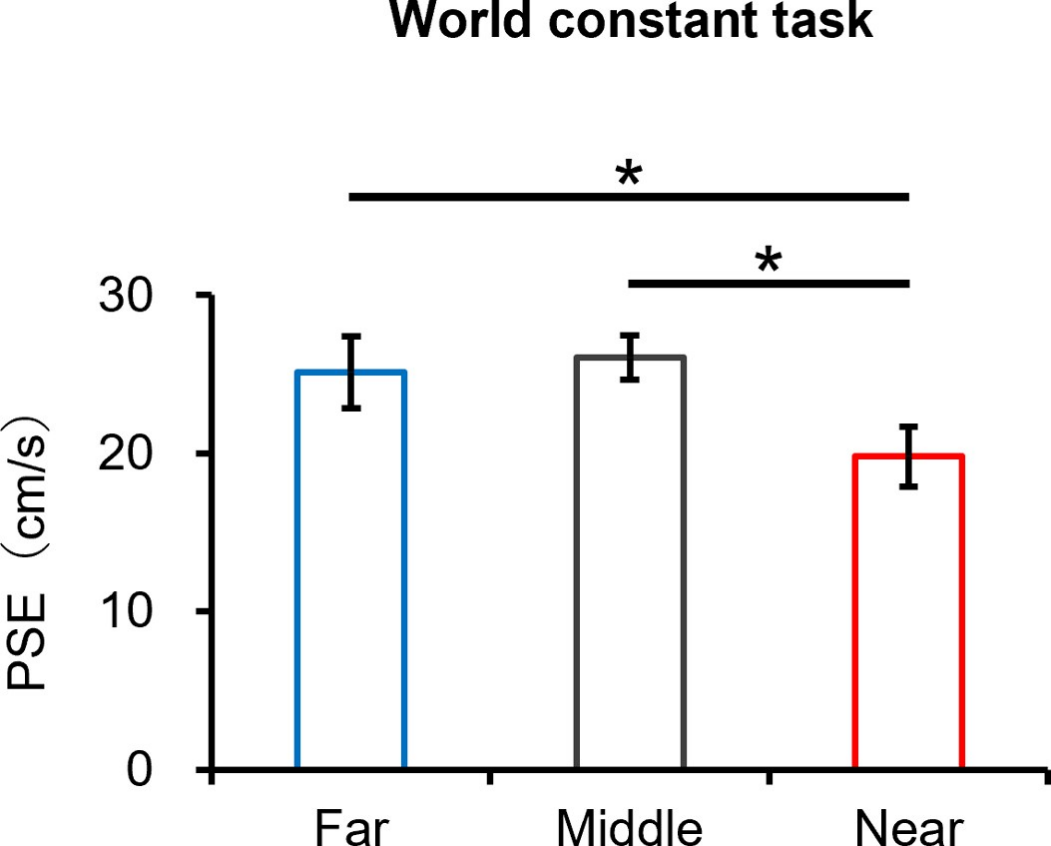
The comparison of the PSE between the far, middle and near conditions in the world constant task. Error bars indicate the standard deviation. *: *p* < 0.05.

We hypothesized that if speed perception relies on real-world motion, there would be no difference in speed perception between conditions for the world constant task, and we would perceive a target faster in order of far, middle and near conditions for the retinal constant task. In contrast, we hypothesized that if speed perception relies on retinal motion, we would perceive a target faster in order of the near, middle and far conditions for the world constant task, and there would be no difference in speed perception between conditions for the retinal constant task. For the world constant task, a typical example of psychometric functions is shown in Fig 4. The mean values (± SD) of the PSE in the far, middle and near conditions were 25.4 ± 1.7, 25.5 ± 1.0 and 19.4 ± 2.0, respectively (Fig 5). The paired t-test showed that the PSE in the near condition was significantly lower than that in the far and middle conditions (*t*(8) = 5.61, *p* < 0.05, *r* = 0.89 and *t*(8) = 7.20, *p* < 0.05, *r* = 0.93, respectively). These results imply that the near condition was perceived faster than the far and middle conditions.

**Fig 6:**
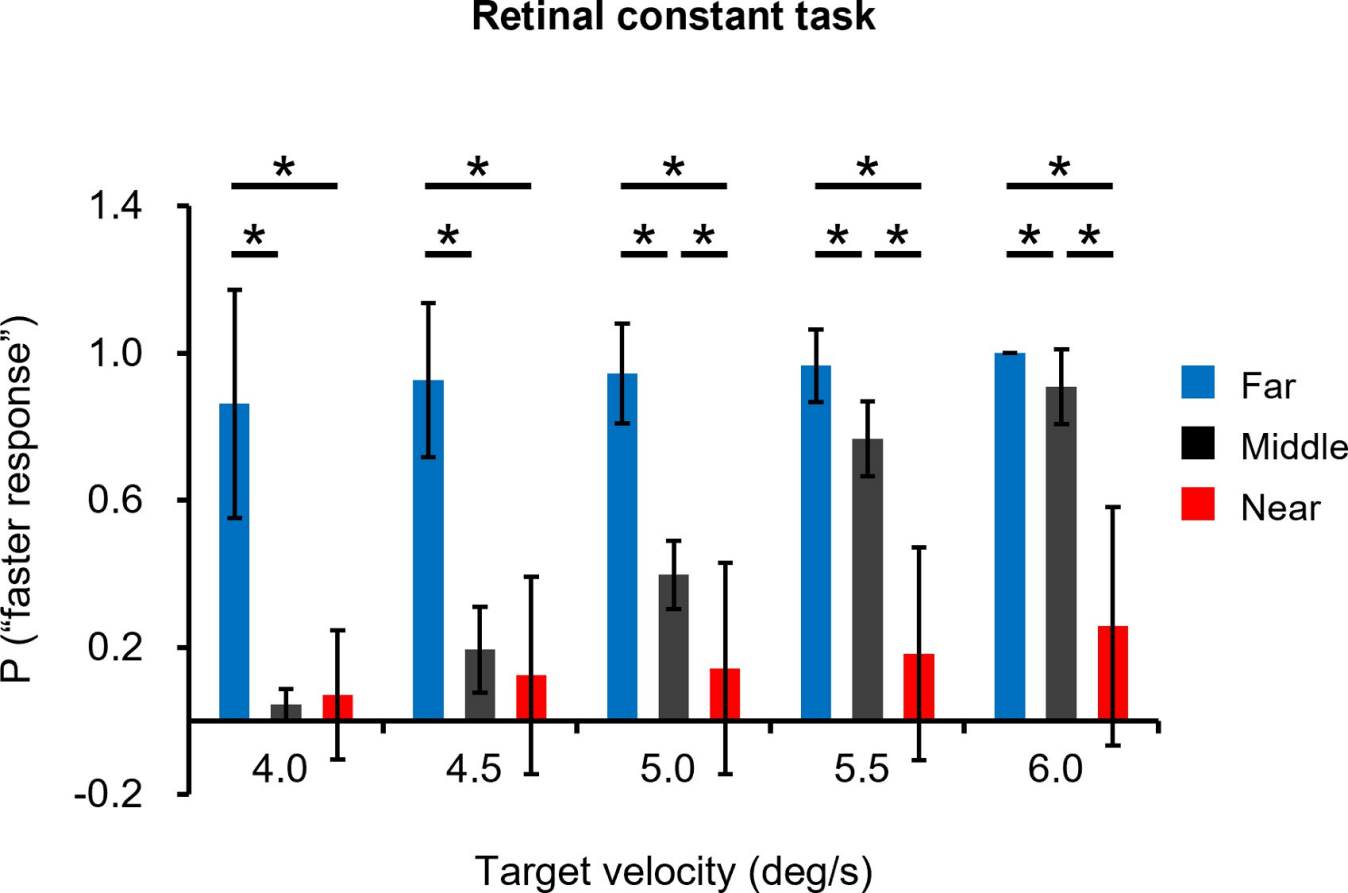
The comparison of the probabilities between the far, middle and near conditions in the retinal constant task. Colors indicate the three different initial positions of the target from the participants: blue, black and red bars are the far, middle and near conditions, respectively. Error bars indicate the standard deviation. *: *p* < 0.05.

For the retinal constant task, there were no significant models from the binomial logistic regression analysis for the far and near conditions because the velocities of all comparison stimuli were clearly different from the standard stimuli. Therefore, we compared the probability of responding that the comparison stimulus was faster than the standard stimulus, in which the target always moves at 4.5 deg/s in the middle condition, between each condition (Fig 6). These results indicate that the far condition is perceived faster than the middle and near conditions. The paired t-test showed that the probability of the far condition was significantly higher than that of the middle and near conditions at target velocities of 4.0 and 4.5 deg/s (all *p*’s < 0.05). The paired t-test also showed that the probability was significantly higher in order of the far, middle and near conditions at target velocities of 5.0, 5.5 and 6.0 deg/s (all *p*’s < 0.05). These results indicate that visual target speed is perceived faster in order of the far, middle and near conditions.

The above results suggest that speed perception for a visual target moving from far to near is influenced by real-world motion rather than by retinal motion. However, speed perception could be influenced by retinal motion or other factors when a target moves close to the observer.

## Experiment 2: Comparison between the fixation and eye movements tasks

### Methods

Eleven participants (10 men and 1 woman; 22.8 ± 1.7 years old) volunteered to take part in this experiment. They reported having normal or corrected to normal vision and no known neurological or oculomotor disorder. This study was conducted in accordance with the Declaration of Helsinki, and all protocols were approved by the Research Ethics Committee at the Faculty of Health and Sport Sciences, University of Tsukuba. Written informed consent was obtained from all subjects before their participation.

**Fig 7:**
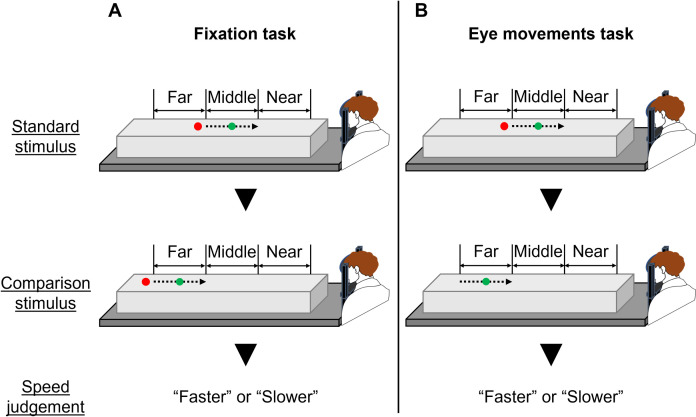
A two-alternative forced-choice paradigm. Fixation task (A): a green circle (visual target) moved from far to near at constant velocity in the real world and participants kept their eyes on the red circle (fixation point) in both standard and comparison stimuli. Eye movements task (B): a visual target moved from far to near at constant velocity in the real world, and participants kept their eyes on the fixation point in the standard stimulus and pursued the target with their eyes in the comparison stimulus. For both speed perception tasks, the participants compared the target speed between the standard and comparison stimuli.

The experimental setup in experimental 2 was identical with that in experimental 1 (Fig 1). To attain the second purpose, we conducted a fixation task and an eye movement task using a 2AFC paradigm. In both tasks, participants kept their eyes on the fixation point when presented with the standard stimulus. The target initial position and target velocity for the fixation task were identical with the world constant task in experiment 1 (Fig 3A–3C). In the fixation task (Fig 7A), participants kept their eyes on the fixation point when presented with the comparison stimulus. The trials, in which eye movements occurred during the fixation task, were excluded. In the eye movements task (Fig 7B), participants tracked a target with their eyes when presented with the comparison stimulus. Participants performed each task on two separate days considering the effects of their fatigue and burden.

Methods for calculating the PSE and convergence eye movements were identical with experimental 1. We also calculated the mean velocity of convergence eye movements from 100 to 400 ms after the motion onset, which allowed us to quantify the speed of the initial oculomotor response, based on potential differences in latency, acceleration, or peak velocity [11]. Furthermore, we calculated the mean retinal slip velocity, which was defined as the difference between target and eye velocities, during convergence eye movements from 100 to 400 ms after the motion onset.

We analyzed the relationship between the probability, which a participant indicated “Faster” in the 2AFC paradigm, and the mean retinal slip velocity during convergence eye movements in three different conditions for eye movement task using the Pearson product-moment correlation coefficient. The other statistical analysis was identical with experimental 1.

### Results

**Fig 8:**
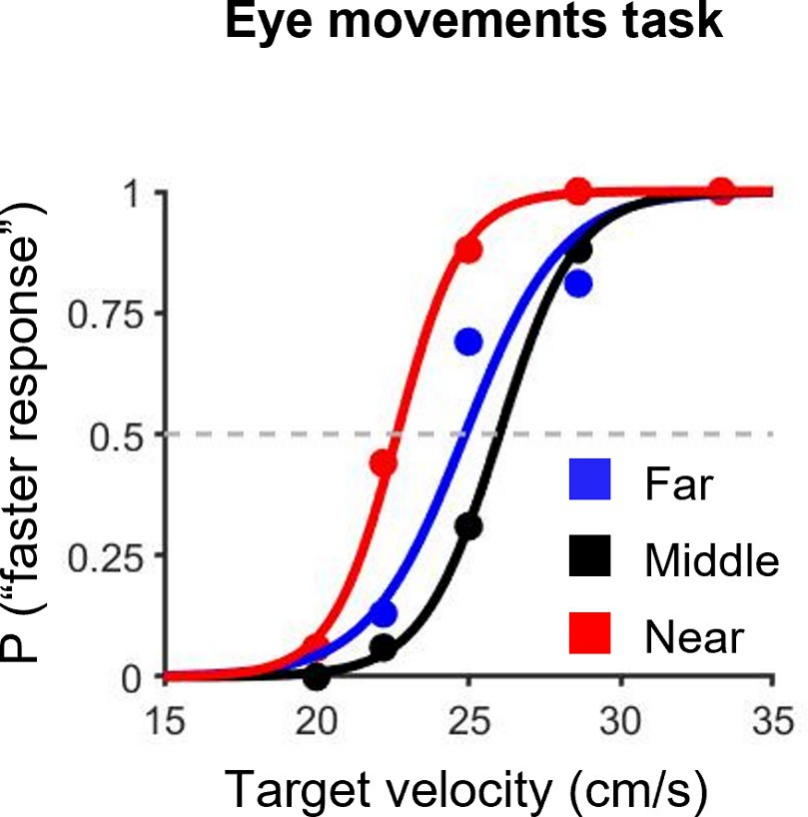
Typical examples of psychometric functions for the eye movements tasks. The blue, black and red lines indicate the far, middle and near conditions, respectively.

**Fig 9:**
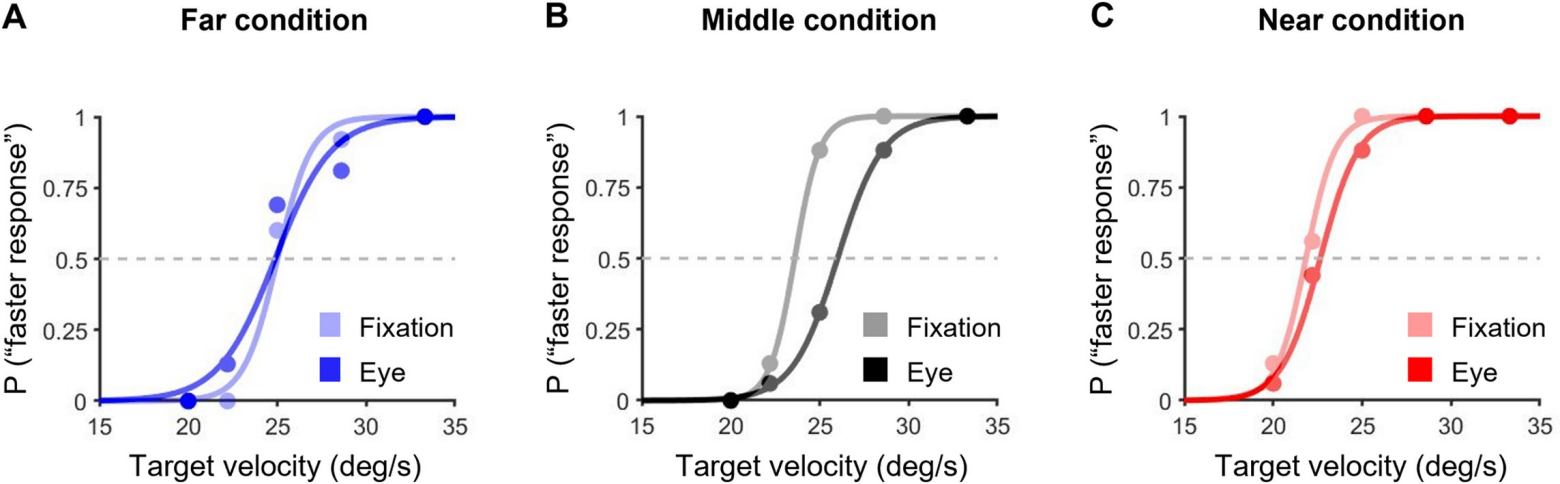
Comparisons of psychometric functions between the fixation and eye movements tasks in the far (A), middle (B) and near (C) conditions. The light colored and dark colored lines indicate the fixation and eye movement tasks, respectively. The blue, black and red lines indicate the far, middle and near conditions, respectively.

Typical examples of psychometric functions for the eye movements task are shown in Fig 8. Fig 9 indicates typical examples of psychometric functions for the comparison between the fixation and eye movement tasks in each condition.

**Fig 10:**
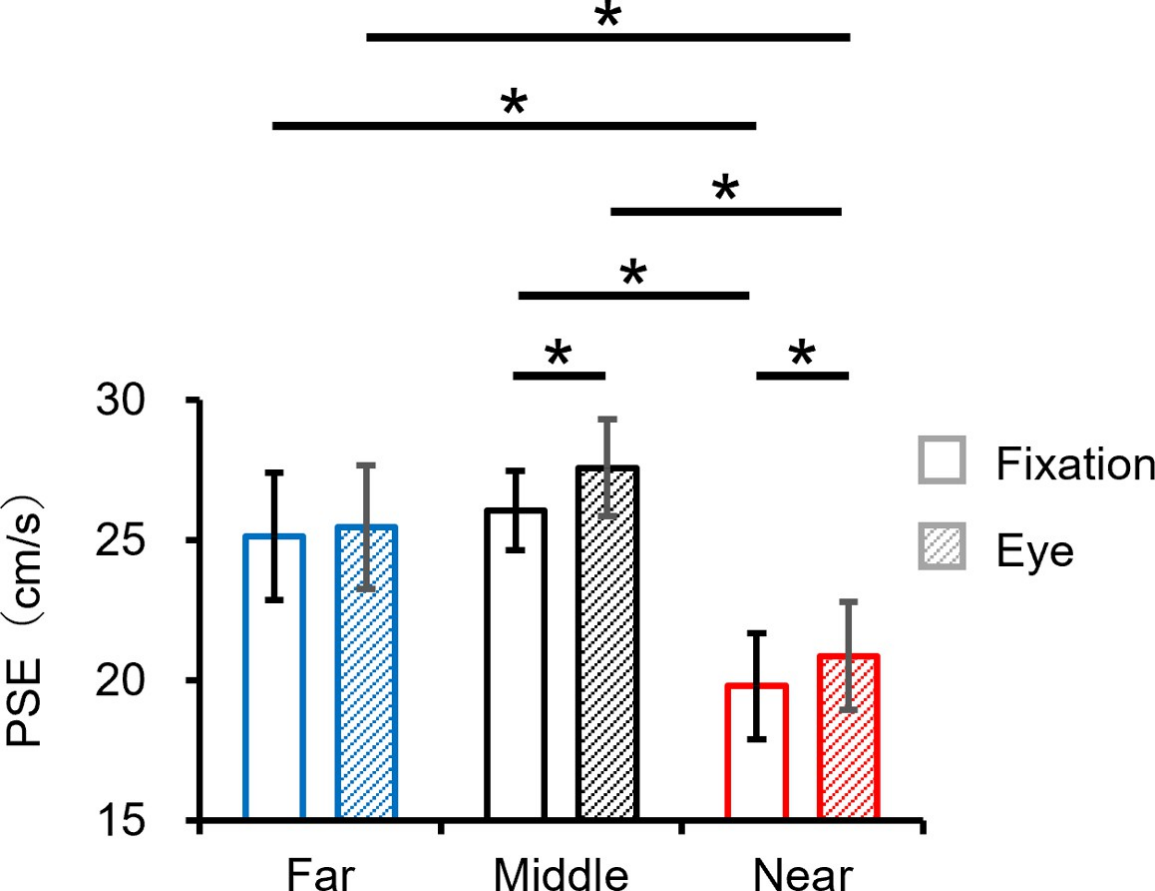
The comparison of the PSE between the far, middle and near conditions in the fixation and eye movements tasks. Error bars indicate the standard deviation. *: *p* < 0.05.

For the fixation task, the mean values (± SD) of the PSE in the far, middle and near conditions were 25.1 ± 2.2, 25.9 ± 1.5 and 19.7 ± 1.8, respectively (Fig 10). For the eye movements task, the mean values (± SD) of the PSE in the far, middle and near conditions were 25.5 ± 2.1, 27.5 ± 1.7 and 20.9 ± 1.9, respectively (Fig 10). The paired t-test showed that the PSE in the near condition in each task was significantly lower than that in the far and middle conditions in each task (near and far condition in the fixation task: *t*(10) = 5.13, *p* < 0.05, *r* = 0.85; near and middle condition in the fixation task: *t*(10) = 8.34, *p* < 0.05, *r* = 0.94; near and far condition in the eye movements task: *t*(9) = 4.05, *p* < 0.05, *r* = 0.80; near and middle condition in the eye movements task: *t*(9) = 7.36, *p* < 0.05, *r* = 0.93). The paired t-test also showed that the PSE values in the middle and near conditions for the fixation task were significantly lower than that in the eye movements task (middle condition: *t*(10) = -3.93, *p* < 0.05, *r* = 0.78; near condition: *t*(10) = -3.12, *p* < 0.05, *r* = 0.72). These results imply that the near condition was perceived faster than the far and middle conditions in both tasks and the fixation tasks in the middle and near conditions were perceived faster than the eye movements tasks. Therefore, these results suggest that the effects of real-world and retinal motion on speed perception for motion in depth are similar in tracking a visual target with convergence eye movements and fixation. However, these results also indicate that convergence eye movements alter the speed perception of a visual target moving from far to near.

**Fig 11:**
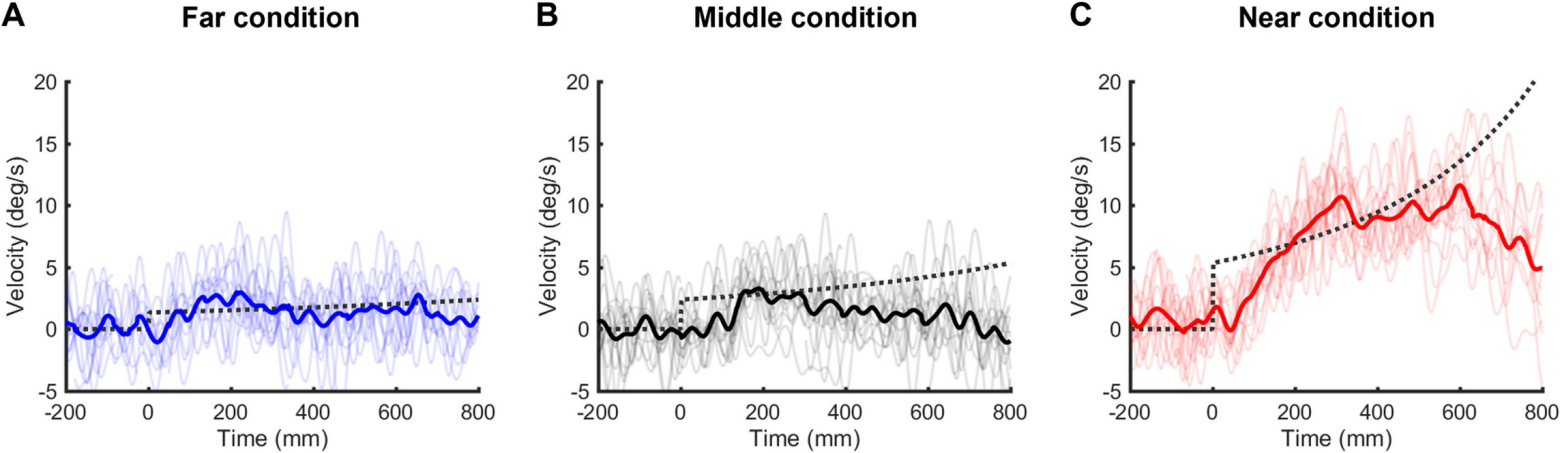
Typical examples of the velocity of convergence eye movements at target velocity of 25.0 cm/s for the eye movement task in the far (A), middle (B) and near (C) conditions. The thin and thick lines indicate the velocity for each trial and the mean velocity, respectively. The dotted lines indicate target velocity.

Fig 11 shows typical examples of the velocity of convergence eye movements at target velocity of 25.0 cm/s in each condition for the eye movement task. Table 1 indicates the mean values (± SD) of the mean eye velocity at each target velocity in three different conditions for the eye movement task.

**Table 1:** Mean values (± SD) of the mean velocity of convergence eye movements from 100 to 400 ms after the motion onset at each target velocity in three different conditions.

	20.0 cm/s	22.2 cm/s	25.0 cm/s	28.6 cm/s	33.3 cm/s
Mean velocity (deg/s)					
Far	1.51 ± 0.35	1.54 ± 0.29	1.80 ± 0.39	2.00 ± 0.47	2.36 ± 0.49
Middle	2.49 ± 0.71	2.69 ± 0.50	2.93 ± 0.84	3.27 ± 0.87	3.69 ± 1.16
Near	5.08 ± 1.75	5.83 ± 1.76	6.40 ± 2.69	6.84 ± 2.75	7.64 ± 3.35

20.0 cm/s, 22.2 cm/s, 25.0 cm/s, 28.6 cm/s and 33.3 cm/s indicate five different target velocities. Far, Middle and Near indicate three conditions.

The correlation analysis showed that there were no significant relationships between the probability that participants indicated “Faster” in the 2AFC paradigm and the mean eye velocity at 25 cm/s of target velocity in all conditions (far condition: *r* = 0.34, *p* = 0.72; middle condition: *r* = -0.15, *p* = 0.69; near condition: *r* = 0.30, *p* = 0.37).

## Discussion

### The effect of the difference between retinal and real-world motions on speed perception

For the world constant task, the target speed in the near condition was perceived as faster than that in the far and middle conditions. In contrast, for the retinal constant task, the target speed was perceived faster in order of the far, middle and near conditions. Thus, the speed of a visual target moving from far to near could be perceived as the speed of the real world under middle and far conditions. It is also suggested that the target speed in the near condition was perceived using other factors rather than real-world motion.

It is possible that the participants in this study primarily used real world motion to perceive the target speed. Lopez-Moliner and colleagues (2003) have demonstrated that participants did not utilize the acceleration of the visual target to motion in depth when estimating the time to contact, which largely supports our results [4]. However, the participants did not always use real-world motion to perceive target speed, but they used some cues other than real-world motion when the target position was close to the observer. We list four factors as such cues.

The first factor is retinal motion, which depends on the vergence angle. The visual target speed on the retina exponentially increases as the real-motion target approaches the peripersonal space, which is defined as the region of space immediately surrounding our bodies in which we can grasp and manipulate objects [12]. This was the case even if the target moves from far to near at constant speed in the real world. Thus, the speed perception in the near condition may be more dependent on retinal motion than real-world motion per se. Lee and colleagues (2019) have also reported that there was no difference in speed change discrimination between retinal and world constant speed [3]. Therefore, we may use both retinal and real-world motion to perceive visual motion speed for a target moving from far to near.

The second factor is the near-response cells, which are specific cells activated when animals view a near target [13, 14]. Thus, signals from these particular cells might influence the determination of visual target speed in the near condition for the world constant task, even though a causal relationship between the near-response cells and speed perception is unaccountable.

The third factor is looming. The looming is one of the depth cues and represents that the visual target image on the retina exponentially increases as the target approaches the observer [15]. In previous studies, it has been reported that the looming cues lead to subjective time dilation [16–18]. These observations are not identical to our results. Lee and colleagues (2020) have also demonstrated that the speed change in looming stimuli is not used for speed change discrimination [19]. Therefore, in this study, the looming cue is not necessarily imperative for visual target speed perception.

The fourth factor is a threatening stimulus. Previous studies have shown that the observer’s brain activity changes when a threatening stimulus is presented to one. For example, it has been reported that the activity of the ventromedial prefrontal cortex is specifically modified when the observer is exposed to dangerous and emotionally negative stimuli [20–26]. Low and colleagues (2008) have also revealed that late positive event-related potentials increase when the observer is close to danger [27]. Other studies have demonstrated that a threatening stimulus approaching the observer modulates perception [28–31]. Furthermore, the time-to-contact (TTC) estimation of a visual target approaching the observer was determined to be shorter than the actual TTC by the threat [28, 30]. This shortened TTC estimation is similar to our results that a visual target was perceived faster in the near condition for the world constant task. Furthermore, Previous studies have shown that animals and humans take evasive or defensive actions when objects approach a direct collision course [32–35]. Therefore, they may have perceived the visual target faster in the near condition than in the middle or far condition for the world constant task as a result of perceiving the potential danger of the target approaching the observer in the near condition.

This study revealed differences in the effects of retinal and real-world motions on speed perception for motion in depth. Further study is necessary because we have not verified the effect when the target moves from near to far. We speculate that if we conduct the same experiment as this study when the target moves from near to far, speed perception would be different from the results obtained in the near condition of this study. That is because the effect of the threatening stimulus, which is one of the factors affecting speed perception in the near condition, is removed. Therefore, it is necessary to examine the effect of different depth motion directions on speed perception in retinal and real-world motion in the future.

### The effect of convergence eye movements on speed perception

For the eye movement task, the target speed in the near condition was perceived faster than that in the far and middle conditions as well as the results of the world constant task. Thus, the speed of an approaching visual target could be perceived as the speed of the real-world under the middle and far conditions. It is also suggested that target speed in the near condition was perceived based on retinal motion, convergence eye movement or other factors described above, rather than real-world motion. Therefore, the effects of retinal and real-world motion on speed perception for motion in depth could be similar with or without convergence eye movements. However, a visual target in the middle and near conditions for the fixation task was perceived faster than that for the eye movements task. Our results also showed that there are significant positive relationships between the probabilities that participants indicated “Faster” in the 2AFC paradigm and the retinal slip velocities in the middle and near conditions for the eye movement task. Nefs and Harris (2007) have shown that a visual target moving in depth is perceived slower when observers pursue it with their eyes than when they keep their eyes stationary [2], which is consistent with our results. It has also been reported that in planar motion, a moving target is perceived as slower when tracking the target with the eyes than when fixating on a stationary spot [1]. Some previous studies have demonstrated that extra-retinal signals are used when determining the visual target speed in the depth direction [2, 6, 36, 37]. It has also been suggested that the combination of retinal and extra-retinal signals leads to imprecise speed perception of a visual target than retinal signal alone, since the sum of retinal with extra-retinal signals could generate the internal noise [38]. Thus, pursuing a target with eye movements could alter the speed perception relative to the fixation. However, the results of the present study indicate that speed perception was slower during convergence eye movements compared to fixation in the middle and near conditions, but no such difference was observed in the far condition. This result could be due to that the target velocity in the far condition was much lower than that in the middle and near conditions (Fig 3). Therefore, it is suggested that extra-retinal signals influence speed perception and that convergence eye movements could alter the speed perception of a visual target approaching the observer when the target velocity is higher or is close to the observer.

The results of the present study indicate that there were no relationships between the probability that participants indicated “Faster” in the 2AFC paradigm, and the mean velocity of convergence eye movements. It rather seems to be the case that variations in eye velocity are not directly related to perceived speed, although pursuing a target may cause some perceptual bias compared with motion perception during fixation. The Bayesian model implemented by Freeman and colleagues (2010) suggests that a visual target motion is important for perceiving head-centered velocity [38]. It has also been shown that a visual target for motion in depth is predicted by the combination of retinal slip and eye velocity [6, 37]. Thus, speed perception may not be dependent on eye velocity per se. Convergence eye velocity in the initial phase may not always affect speed perception when a visual target approaches. In contrast, for planar motion, previous studies have suggested that the initial phase of the target motion is important for determining speed and duration [5, 39, 40]. These differences might be related to the difference between planar and depth motions on the retina. Since the visual target for motion in depth does not move at constant velocity on the retina even if the target moves at constant velocity in the real world, the speed perception of motion in depth may not always be associated with the initial phase of vergence eye movements. The relationship between speed perception of motion in depth and vergence eye movements needs to be further verified.

## Conclusions

We aimed to reveal the effects of retinal and real-world motion on speed perception of a visual target moving from far to near, and to verify the influence of eye movements on both motion signals in judging speed in depth. Our results demonstrate that speed perception of a visual target approaching the observer is dependent on real-world motion when a target position is relatively far from the observer. In contrast, when a target position is close to the observer, speed perception is related to retinal motion or other factors rather than real-world motion. Thus, it is suggested that when the visual target moves from far to near, the effects of real-world and retinal motion on speed perception are different depending on the initial target position. Moreover, we found that stimulus speed was perceived faster in the near condition than in the middle and far conditions, regardless of whether the observer was fixating on a stationary spot or tracking a target with eye movements. Therefore, it is suggested that the effect of real-world and retinal motion on speed perception when the target moves toward the body could be similar when fixating on a stationary spot and tracking a target with convergence eye movements.
